# SensorDrop: A system to remotely detach individual sensors from wildlife tracking collars

**DOI:** 10.1002/ece3.10220

**Published:** 2023-07-04

**Authors:** K. Rafiq, R. G. Appleby, A. Davies, B. Abrahms

**Affiliations:** ^1^ Department of Biology, Center for Ecosystem Sentinels University of Washington Seattle Washington USA; ^2^ Botswana Predator Conservation Maun Botswana; ^3^ Centre for Planetary Health and Food Security Griffith University Brisbane Queensland Australia; ^4^ Wild Spy Pty Ltd Brisbane Queensland Australia; ^5^ Arribada Initiative Cheshire UK

**Keywords:** animal‐borne sensors, bio‐logging, conservation technology, drop‐off, open‐source hardware, radiocollar

## Abstract

The growing diversity of animal‐borne sensor types is revolutionizing our understanding of wildlife biology. For example, researcher‐developed sensors, such as audio and video loggers, are being increasingly attached to wildlife tracking collars to provide insights into a range of topics from species interactions to physiology. However, such devices are often prohibitively power‐intensive, relative to conventional wildlife collar sensors, and their retrieval without compromising long‐term data collection and animal welfare remains a challenge. We present an open‐source system (SensorDrop) for remotely detaching individual sensors from wildlife collars. SensorDrop facilitates the retrieval of power‐intensive sensors while leaving non‐resource‐intensive sensors intact on animals. SensorDrop systems can be made using commercially available components and are a fraction of the cost of other timed drop‐off devices that detach full wildlife tracking collars. From 2021 to 2022, eight SensorDrop units were successfully deployed on free‐ranging African wild dog packs in the Okavango Delta as part of audio‐accelerometer sensor bundles attached to wildlife collars. All SensorDrop units detached after 2–3 weeks and facilitated the collection of audio and accelerometer data while leaving wildlife GPS collars intact to continue collecting locational data (>1 year), critical for long‐term conservation population monitoring in the region. SensorDrop offers a low‐cost method to remotely detach and retrieve individual sensors from wildlife collars. By selectively detaching battery‐depleted sensors, SensorDrop maximizes the amount of data collected per wildlife collar deployment and mitigates ethical concerns on animal rehandling. SensorDrop adds to the growing body of open‐source animal‐borne technologies being utilized by wildlife researchers to innovate and expand upon data collection practices and supports the continued ethical use of novel technologies within wildlife studies.

## Graphical abstract

The retrieval of specific animal‐borne sensors without compromising long‐term data collection and animal welfare remains a challenge. In this manuscript, we present SensorDrop: a system for remotely detaching auxiliary animal‐borne sensors from wildlife collars while leaving main collar units attached. By selectively detaching battery‐depleted sensors, SensorDrop maximizes the amount of data collected per wildlife collar deployment and mitigates ethical concerns on animal rehandling.

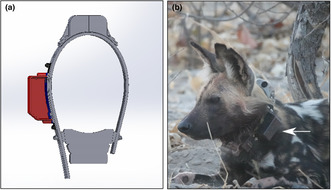

## 1 INTRODUCTION

Advancements in animal‐borne technologies are revolutionizing our understanding of species biology in the wild. In the past decade, the diversity of sensor types used in wildlife studies and the questions they are helping to answer have rapidly diversified. For example, audio recorders have helped quantify the hunting performance of predators (Studd et al., 2021), video recorders are giving new insights into the cues species use to navigate (Yoshino et al., 2020), and accelerometers are elucidating the energetic consequences of animal behaviors (Nickel et al., 2021). While the specific attachment mechanisms used to deploy sensors onto wildlife vary with species‐ and locale‐specific characteristics, one commonly used mechanism for many medium to large terrestrial species is the wildlife tracking collar (herein wildlife collars) (for an overview see Rafiq, Pitcher, et al., 2021).

Wildlife collars are commonly used in wildlife ecology and conservation for studying the relationships between species and their environments. Collar types range from those allowing for triangulation of individuals using non‐data logging transmitters (e.g., radio transmitters), detectable within limited ranges by specialist telemetry equipment, to more advanced global positioning system (GPS) logging collars with the capacity to collect, and optionally transmit, locational data throughout the day (Kays et al., 2015; Rafiq, Pitcher, et al., 2021). Yet while conventional collars have no doubt enhanced our capacity to understand animal behavior in the wild (Kays et al., 2015; Wilmers et al., 2015), innovations in data logging capabilities have largely been limited to a few commonly used sensor modalities (e.g., GPS and accelerometer) by commercial manufacturers. In response, researchers are increasingly adapting or developing new technologies for use in animal‐borne studies, which has increased the diversity of sensor types available for wildlife research and enhanced the field's research capacity (e.g., Hernandez et al., 2018; McGregor et al., 2015; Rafiq, Appleby, et al., 2021).

Two common barriers to the uptake of novel animal‐borne technologies are power requirements (with higher requirements typically necessitating shorter sensor deployment durations) and unit retrieval. Fundamental to many studies utilizing wildlife collars are the spatial data they provide, with additional auxiliary sensors commonly used to provide further context to animal movements (Kays et al., 2015). However, unlike GPS tags, for example, which are often both relatively power‐un‐intensive, due to the type of data they collect, and optimized for power efficiency, due to how long such sensors have been used in wildlife studies, new sensor technologies can have relatively high‐power requirements (De La Rosa, 2019; Wijers et al., 2018). In some cases, for example, new wildlife sensors are adapted from technologies developed for industries where power efficiency is less of a consideration (such as sports action cameras, e.g., McGregor et al., 2015). Furthermore, even in cases where sensors are specifically designed for wildlife studies, the characteristics of the data they collect may necessitate relatively high‐power requirements (such as audio data, e.g., Wijers et al., 2018).

As a consequence, when attaching new sensor technologies to wildlife collars, researchers must frequently navigate power limitations by either (i) recovering battery‐depleted sensors shortly after deployment through animal rehandling or remote collar detachment or (ii) leaving battery‐depleted sensors on wildlife until all collar sensors have finished collecting data. In the former, researchers have quicker access to data, and animals carry the additional weight of battery‐depleted sensors for less time. However, there are ethical and welfare implications of (i) rehandling animals, which often induces stress and can involve the use of immobilization drugs that carry inherent risks (Kreeger & Arnemo, 2018; Meyer et al., 2008; Soulsbury et al., 2020), and (ii) deploying sensors for such short deployment durations, particularly in environments where multiple collaborating research partners have different data needs (e.g., long‐term monitoring of populations versus short‐term research projects). In contrast, by leaving depleted sensors on wildlife for longer, researchers can maximize the overall data collected per deployment effort, but animals must carry the additional weight of non‐functional sensors, and researchers may need to wait multiple additional months to retrieve the data. Currently, no openly available system exists that allows supplemental sensors to be remotely detached from wildlife collars, with the best alternative involving adapting full collar drop‐off mechanisms (e.g., from Vectronic Aerospace, Telonics, or Lotek Wireless) in ways technically challenging for the skillsets of many ecologists (see: wildlabs.net/discussion/drop‐pods‐collars).

Here we present an innovative open‐source system (SensorDrop) for remotely detaching auxiliary animal‐borne sensors from wildlife collars while leaving the main collar units attached. SensorDrop builds upon and extends the functionality of existing open‐source detachment mechanisms (Rafiq et al., 2019). Specifically, SensorDrop uses the core electronics of Rafiq et al. (2019) within a novel hardware design (presented below) to detach specific sensor bundles from wildlife collars and represents >800 h of additional development and field‐testing time. SensorDrop is simple to use, is compatible with a wide range of sensor types, and is a fraction of the cost of comparable commercial drop‐off mechanisms that detach full collars. Here, we provide a detailed system components summary and a case study of its use on free‐ranging African carnivores to demonstrate the utility of SensorDrop in real‐world settings.

## 2 SYSTEM OVERVIEW AND COMPONENTRY

**FIGURE 1:**
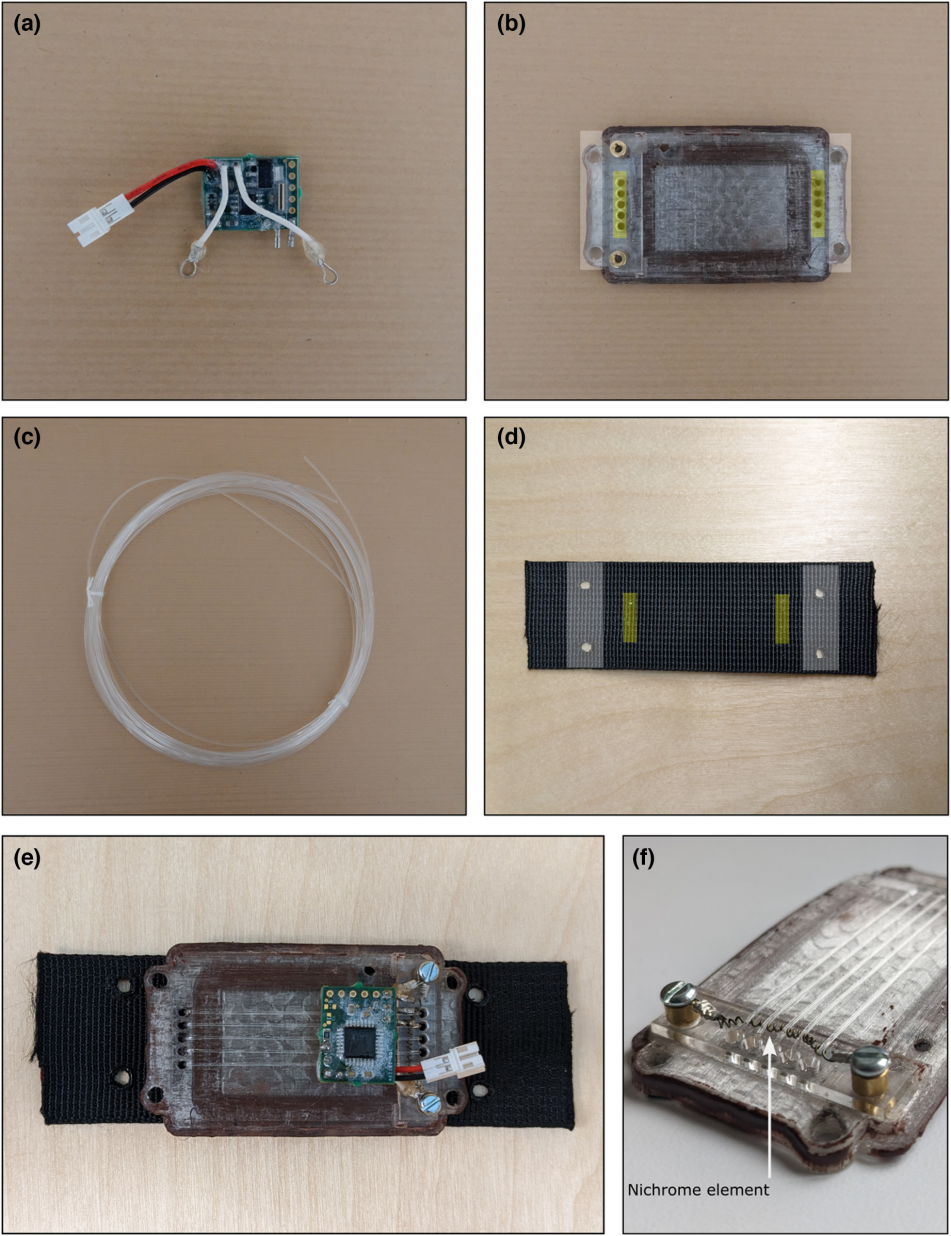
Main SensorDrop components. (a) OpenDrop Printed Circuit Board. (b) Drop‐off plate. Holes highlighted in white are used to screw the drop‐off plate to the user‐designed sensor housing. Holes highlighted in yellow are used to connect the drop‐off plate to the nylon webbing. (c) Nylon line. (d) Nylon webbing used to connect drop‐off plate to wildlife collar. Holes highlighted in yellow are used to connect the Drop‐off plate to the webbing. Holes highlighted in white are used to connect the webbing to the wildlife collar. (e) Assembled SensorDrop system. (f) Close‐up of the coiled nichrome element and nylon lines.

SensorDrop is composed of four key components that together attach to a sensor housing of the user's design: (i) an OpenDrop printed circuit board (PCB); (ii) a drop‐off plate; (iii) nylon line; and (iv) nylon webbing (Figure 1). The total component cost of SensorDrop ranges from $25.36 to $60.77 per device, with costs decreasing as more units are needed due to decreasing part costs (Table S1).

The OpenDrop PCB is composed of a timer circuit that, at a user‐determined time, allows electrical current to flow to a nichrome element for a short pulse (~5 s), causing the nichrome element to heat and melt the nylon line securing the drop‐off mechanism to the collar (see Rafiq et al., 2019). Within SensorDrop, the OpenDrop PCB is powered by a Tadiran 220 mAH 20C lithium‐polymer battery (expected deployment duration of approximately 3 months) and connected to a coiled nichrome element, which heats to temperatures exceeding 150°C. Several factors interact to control the heat of the nichrome element, mainly the lengths of the connecting wires and the gauge and format (i.e., coiled or straight) of the nichrome element. Coiling the nichrome, for example, has the effect of intensifying the heat generated as current runs through the wire. Moreover, as you increase the length of any of the connecting wires, the relative resistance within the circuit increases and the maximum temperature the nichrome element reaches decreases. In contrast, decreasing the length of the connecting wires will achieve the opposite and can, if the nichrome element contacts other components of the sensor housing, lead to open flames. As such, we refer users to the configurations provided within the SensorDrop online repository as an initial starting point, and we encourage thorough testing in safe and controlled environments before any field deployments. Additionally, for safety, nichrome elements should remain fully contained within sensor housings to avoid contact with the natural environment (animals and vegetation). Full details on programming the OpenDrop PCB can be found in Rafiq et al. (2019) and the associated online repository.

The SensorDrop drop‐off plate connects the main sensor housing that the user wishes to detach, the nylon webbing, and the OpenDrop PCB (Figure S1). Files for 3D printing, CNC‐milling, and modifying previously used sensor housings compatible with SensorDrop can be found within the SensorDrop online repository. Depending on species and location‐specific stressors expected to be placed on the wildlife collars, the drop‐off plate can be 3D printed and coated with finishing resin for water resistance (e.g., X3D Finishing Epoxy Resin) or CNC‐milled for greater strength. The plate contains two parallel rows of five holes at opposing ends, with a polycarbonate strip (2 mm thickness) attached across one set of holes. The nichrome element is suspended across the polycarbonate strip using brass spacers in order to avoid direct contact between the nichrome and drop‐off plate, which would otherwise decrease the final temperature of the nichrome element and could lead to failure in melting the nylon line (Figure 1).

Up to five lines of high tensile strength monofilament nylon line are looped across the suspended nichrome element through the plate holes and through mirroring holes on the nylon webbing in order to attach the drop‐off plate to the nylon webbing with a surgeon's knot. To increase the unit's water resistance, silicon adhesive can be used to fill the plate holes prior to the nylon line being looped through. The appropriate number of nylon lines to use and their tensile strength will depend on the specifics of the study system, particularly the forces expected to be placed on sensors during deployment. Too few or weak lines will result in premature detachment of the unit, for example, with contact from the environment severing lines. In contrast, excessive or overly strong lines can increase the mass of units as larger batteries with higher current capabilities are usually required to sever nylon lines. For example, for social species where sensors can be expected to be pulled, twisted, or bitten by conspecifics, or for species occurring within densely vegetated woodlands where sensors can knock against vegetation, higher numbers of, or higher tensile strength, nylon lines, may be preferred. We recommend increasing the number of nylon lines used to secure the drop‐off plate to the nylon webbing before increasing the tensile strength as thicker lines may require significantly higher current in order for the nichrome element to reach the temperatures able to sever the line. As a general starting point, we recommend using the number and tensile strength of lines that, in sum, match or exceed twice the weight of the mean mass of individuals to be collared (Equation 1). We have successfully tested nylon lines of 68.2 kg (150 lb) (Sakuma Supacast) tensile strength within controlled settings (non‐field deployed) and 22.6 kg (50 lb) (Sunshine Fishing Products) tensile strength nylon lines during field deployments.
(1)

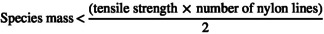




Once the SensorDrop drop‐off plate and nylon webbing are secured together via the nylon line, the drop‐off plate is screwed onto the user‐designed sensor housing and the nylon webbing is bolted or riveted onto the wildlife collar. At the user's pre‐programmed time the nichrome element will heat, melting the nylon line and severing the sensor bundle from the nylon webbing, thereby allowing users to collect detached sensors. Additionally, a VHF transmitter can be embedded into sensor bundles to assist with finding detached units (see case study below), which we would recommend for most use cases given the challenges of finding devices among vegetation. However, for deployments within controlled systems (e.g., captive animals) or species with predictable ranging patterns during deployment (e.g., denning mammals), VHF transmitters may not be required. Upon detachment, the only SensorDrop components left on the wildlife collar are the nylon webbing and nylon lines (weighing ~4 g), thereby minimizing wildlife collar weight from non‐essential supplemental material.

Detailed schematics, design files, and development notes are available within the SensorDrop online repository.

## 3 CASE STUDY

**FIGURE 2:**
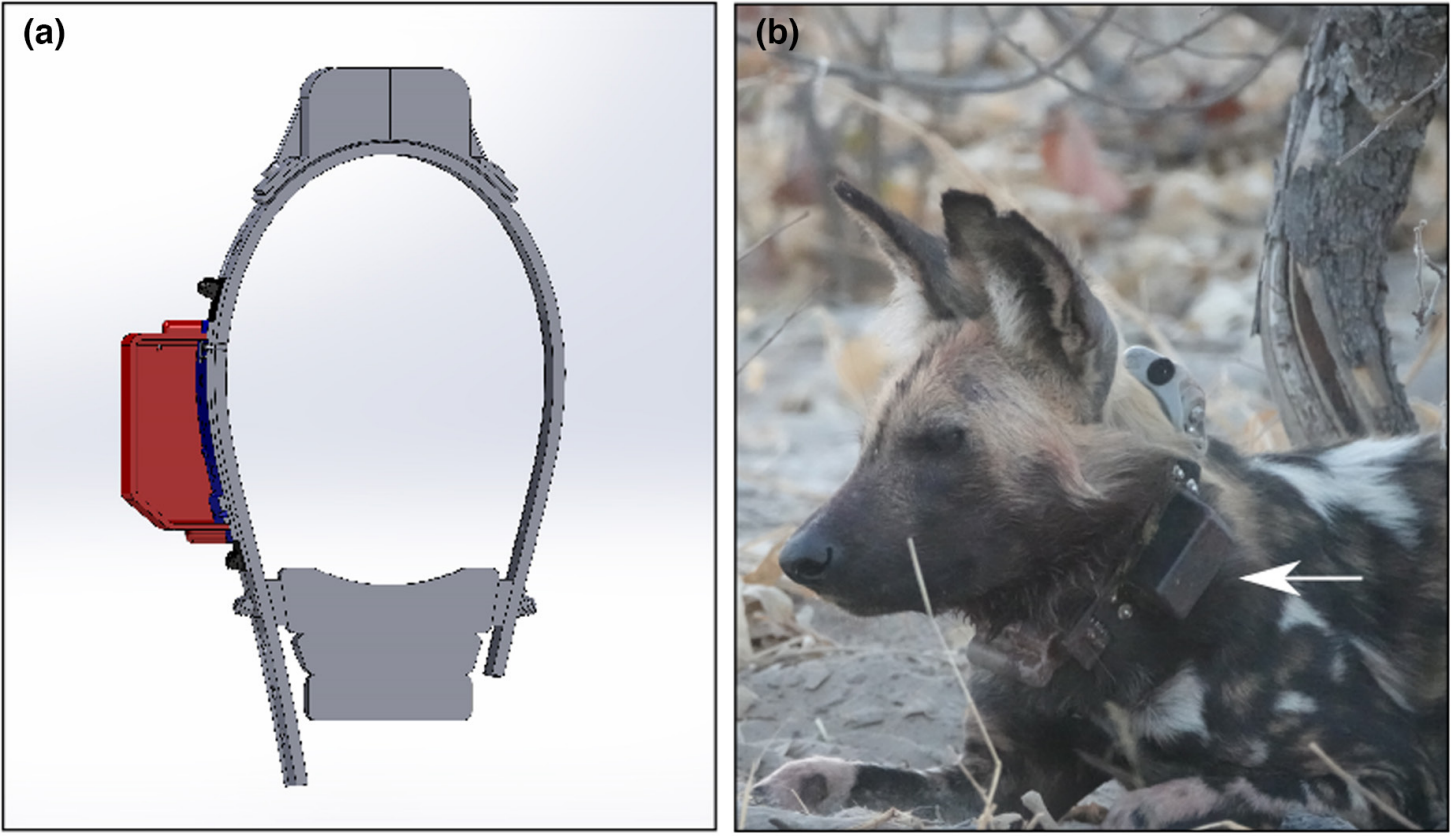
SensorDrop configuration for deployments on African wild dogs in the Okavango Delta. (a) 3D render of SensorDrop housing components used in African wild dog deployments. The commercial Vectronic Vertex Plus 1C collar (factory configuration) is represented in gray. The black and blue components represent the SensorDrop nylon belting and plate, respectively. The red component represents our study‐specific housing designed to contain an audio recorder, accelerometer, and VHF transmitter. For clarity, the SensorDrop PCB and nichrome are not shown. (b) An African wild dog wearing a Vectronic Vertex Plus 1C collar with the SensorDrop unit (arrow).

Between September 2021 and August 2022, we deployed eight SensorDrop detachment mechanisms across individuals in six free‐ranging African wild dog packs in the Okavango Delta (centre coordinates: −19°52′S, 23°63′E). The units were part of a collaborative long‐term monitoring project maintained by Botswana Predator Conservation and a research program led by the University of Washington to investigate the impacts of climate change on carnivore populations. We incorporated SensorDrop into auxiliary sensor bundles, which consisted of an audio recorder (uMoth, Open Acoustic Devices), an accelerometer (AXY‐5, TechnoSmart), and a VHF transmitter (custom unit, Wildlife Materials), that were then attached to Vectronic Vertex Plus 1C wildlife collars (Vectronic Aerospace) (Figure 2). Sensor bundle casings were made of CNC‐milled polycarbonate, and drop‐off plates were constructed from 3D‐printed PLA (design files are available from the online repository). Total wildlife collar weights were 440 g, representing <2% of average African wild dog body weights, with auxiliary sensor bundles (excluding SensorDrop) accounting for 79 g of the total weight and SensorDrop accounting for 26 g. Five strands of monofilament nylon line (22.6 kg/50 lb tensile strength) were used to tie sensors onto nylon webbing (100 × 40 mm) that was then riveted onto the wildlife collars. We fitted collars to anesthetized animals in collaboration with a Botswana‐registered veterinarian using established darting procedures (Hubel et al., 2016, IACUC protocol #4514‐01). We configured all SensorDrop units to remotely detach from wildlife collars 2–3 weeks after sensor deployments, and we visited collared individuals every 2–4 days to collect supplementary data and assess welfare. No ill effects of collar deployments were observed. All SensorDrop integrated units detached within 2–3 weeks following deployments and facilitated the collection of audio and accelerometer data that were available for immediate use, while leaving wildlife GPS collars intact to continue collecting locational data (>1 year), critical for long‐term conservation population monitoring in the region. Seven of the units detached at the pre‐programmed times. One unit detached prematurely after water ingress led to the drop‐off mechanism triggering 6 days early. This was a fail–safe mechanism implemented within the OpenDropOff software to ensure units dropped if SensorDrop battery voltages reached user‐defined critical thresholds, for example, during short circuits caused by water immersion, as in our case (Rafiq et al., 2019). Further investigation of the unit indicated that the water ingress was likely due to the collared individual spending more than anticipated time crossing flooded areas within our study site.

## 4 DISCUSSION

SensorDrop offers a low‐cost method to remotely detach and retrieve individual sensors from wildlife collars. SensorDrop allows researchers to remotely retrieve power and data‐intensive sensors while leaving longer‐lasting sensors intact on animals. This has ethical implications, as it minimizes the total number of sensors carried by individuals, negates the need to recapture animals for sensor removal, and maximizes the amount of data collected per wildlife collar deployment, which is advantageous for studies on wildlife populations involving multiple research agendas with different data needs (e.g., Hubel et al., 2016; Wilson et al., 2013). SensorDrop also allows researchers to mitigate sensor impacts on wildlife by being compatible with user‐designed sensor housings. For example, SensorDrop can be attached to sensor boxes designed to accommodate sensor types in a way that minimizes their impact on species' behaviors, such as with reduced profiles to minimize catching on vegetation.

Another advantage of SensorDrop is its low‐cost and welfare impact compared to alternative sensor retrieval mechanisms. For example, SensorDrop is cheaper than comparable commercial mechanical drop‐off units designed to detach full wildlife collars (e.g., Vectronic Aerospace embedded collar drop‐offs are $480 in 2022). While more affordable bio‐degradable drop‐off mechanisms exist, which typically function by detaching wildlife collars once a bio‐degradable link in the collar decomposes, detachment times can be highly variable and unpredictable, with collars often separating several months before or after when expected (Hellgren, 1988). The low‐cost and component accessibility of SensorDrop, relative to alternative methods, supports ethical innovation of new animal‐borne sensors and increases the accessibility of such tools to species, geographies, and researchers typically underfunded in wildlife research and conservation (dos Santos et al., 2020).

Our case study highlights the applicability of SensorDrop in an endangered large carnivore where the long‐term monitoring needs of a long‐standing NGO must be balanced against shorter‐term research projects by independent collaborating researchers. We expect that the SensorDrop configuration used for African wild dogs would be suitable for similar‐sized species, and we have provided detailed design and assembly information in the online repository. Our case study also illustrates the importance of considering species (and population‐specific) stressors, such as extended periods of water submersion, when designing units. Often species‐ and locale‐specific stressors can place unique combinations of strain on devices that can be difficult to predict and proactively mitigate. We would advise users to pay particular attention to the number, and tensile strength, of the nylon lines used as too many or overly thick lines may necessitate heavier batteries, while too few or weak lines may result in premature detachment. For example, an opportunistic deployment of a SensorDrop unit on an African lion (*Panthera leo*) with the same configuration as that used for African wild dogs led to premature unit detachment after conspecifics pulled the sensor enclosure from the wildlife collar.

The power consumption of units averaged 50 μA during periods of standby and 1–3 A during detachment and required a nominal voltage of ~3.6 V (Rafiq et al., 2019). Thus, with 200 mAH batteries (as used in our case study), we would conservatively estimate maximum deployment times of 133 days before batteries reach critical levels. This assumes we save 20% of the battery for the discharge sequence and equates to a remainder of about 160 mAH for the standby time (160 [remaining capacity]/0.05 [standby draw]/24 [hours in a day] = 133 days). While larger batteries can increase the total possible deployment times of SensorDrop units before detachment, we would advise users to test how larger battery capacities and discharge rates interact to change the temperatures that the nichrome element reaches.

Ultimately, whenever SensorDrop is adapted or deployed into new systems, we would advocate for users to test units in controlled environments thoroughly and to deploy smaller numbers of units during field pilot studies to identify and fix unexpected system‐specific issues. To help, we have included a guide to key SensorDrop device design considerations, potential pitfalls, and resolutions in the device failure sheet in the online repository.

When combined with mature wildlife collar technologies from commercial telemetry brands, SensorDrop provides a low‐risk system for researchers to innovate upon and test new sensors for wildlife research. The growing accessibility of DIY electronics components (such as SparkFun and Arduino) and the narrowing divide between wildlife researchers and technologists (facilitated by the emergence of online interdisciplinary communities such as WILDLABS) has led to a boom in researcher‐developed innovations within the wildlife sciences (e.g., De La Rosa, 2019; Miquel et al., 2022; Rafiq, Appleby, et al., 2021; Wijers et al., 2018). SensorDrop addresses a key barrier to the continued innovation of animal‐borne sensors in that it allows specific animal‐borne sensors to be cost‐effectively remotely detached and retrieved from wildlife collars. Ultimately, SensorDrop adds to the growing body of open‐source animal‐borne technologies being utilized by researchers to innovate and expand upon data collection practices in free‐ranging wildlife, and we believe it will support the continued ethical use of novel technologies within wildlife studies.

## AUTHOR CONTRIBUTIONS

**K. Rafiq:** Conceptualization (lead); investigation (lead); methodology (lead); project administration (lead); writing – original draft (lead); writing – review and editing (lead). **R. G. Appleby:** Methodology (supporting); writing – original draft (supporting); writing – review and editing (supporting). **A. Davies:** Methodology (supporting); writing – original draft (supporting); writing – review and editing (supporting). **B. Abrahms:** Conceptualization (supporting); funding acquisition (lead); methodology (supporting); project administration (supporting); writing – original draft (supporting); writing – review and editing (supporting).

## CONFLICT OF INTEREST STATEMENT

One of the co‐authors (RA) is a financial partner in a company (Wild Spy Pty Ltd) that manufactures animal‐borne devices. Another co‐author (AD) is a director of a non‐profit (Arribada Initiative C.I.C) that develops open‐source conservation technology solutions. However, all necessary information and files for independent manufacture of SensorDrop devices have been provided directly in the manuscript and/or in the associated GitHub repository under the terms of the GNU General Public License v3.0.

## Supporting information


Appendix S1

## Data Availability

Detailed schematics, design files, and development notes are available within the SensorDrop online repository: https://github.com/KasimResearch/SensorDrop.
