# Supplementary material for: SensorDrop: A system to remotely detach individual sensors from wildlife tracking collars
Source: Ecol Evol. 2023 Jul 4;13(7):e10220. doi: 10.1002/ece3.10220 (PMC10318577; doi:10.1002/ece3.10220)
Supplement: Supplementary file 1 — Appendix S1 [file ECE3-13-e10220-s001.zip › supinfo.docx]

**Supplemental information**

**Table 1. Summary of SensorDrop components, approximate prices, and supplier details. Parts are separated into the four main categories of SensorDrop components (boldened and shaded)**

| **Component** | **Qty** | **Supplier Part Number** | **Price per unit (USD)** | **Minimum order price (USD)** | **Material specifications ^a^** | **Website** |
| --- | --- | --- | --- | --- | --- | --- |
| **OpenDrop PCB** |  |  |  |  |  |  |
| PCB | 1 | NA | $10.66 | $ 10.66 (enough for 1 SensorDrop) | NA | Self-producible (see Rafiq et al., 2019) |
| Wires (power and nichrome) | 2 | C2003W-5-ND | $0.14 | $ 0.99 (enough for 7 SensorDrops) | Cable type: hook-up; gauge: 24 AWG; conductor strand: solid; conductor material: copper, annealed tinned | www.digikey.com |
| Ring terminals | 4 | 795-1677 | $0.39 | $ 9.72 (enough for 25 SensorDrops) | Inner ring diameter: 3.68 mm; outer ring diameter; 6.35; contact material: copper; contact plating: tin | [www.rs-online.com](http://www.rs-online.com/) |
| Battery (Turnigy 200 mAH ) | 1 | 9067000498 | $3.49 | $ 3.49 (enough for 1 SensorDrop) | Battery type: lithium polymer; capacity: 200 mAh; voltage 3.7 V; constant discharge rate: 20C | www.hobbyking.com |
|  |  |  |  |  |  |  |
| **Drop-off plate** |  |  |  |  |  |  |
| 3D printed plate | 1 | NA | $6.76 ^b^ | $ 6.76 (enough for 1 SensorDrop) | Printing material: Polylactic Acid (PLA) | www.3dpeople.uk/ |
| Acrylic plate | 1 | B09DP33SP8 | $0.28 | $ 13.99 (enough for 50 SensorDrops) | 2 mm thick acrylic (plexiglass) | [www.amazon.com](http://www.amazon.com/) |
| Brass spacer | 2 | 1772-2606-ND | $2.52 | $ 2.52 (enough for 1 SensorDrop) | Material: brass; thread size: M2.5x0.45 | www.digikey.com |
| Nichrome wire | 1 | B07CHTT73J | $0.02 | $ 7.39 (enough for 300 SensorDrops) | Single strand nichrome; gauge: 28 | [www.amazon.com](http://www.amazon.com/) |
| Spacer screw | 2 | 36-29300-ND | $0.96 | $0.96 (enough for 1 SensorDrop) | Type: machine screw; material: steel; plating: zinc, clear chromate; thread size: M2.5x0.45 | www.digikey.com |
|  |  |  |  |  |  |  |
| **Nylon line** |  |  |  |  |  |  |
| Nylon line | 1 | B0928JDY9Y | $0.03 | $ 7.99 (enough for 250 SensorDrops) | Monofilament fishing nylon, uncoated, and 50 lb (22.6 kg) tensile strength. | www.amazon.com |
|  |  |  |  |  |  |  |
| **Nylon webbing** |  |  |  |  |  |  |
| Nylon webbing | 1 | WN250-BLACK | $0.11 | $ 0.89 (enough for 8 SensorDrops) | Weave nylon webbing | [www.profabrics.co.uk](http://www.profabrics.co.uk/) |
| Total SensorDrop price |  |  | $ 25.36 ^c^ |  |  |  |

*^a^ We provide material specifications for users who want or need to purchase alternative parts than those suggested. Part recommendations are based on component specifications during African wild dog deployments. Other materials/specifications may also be suitable.*

*^b^Price varies significantly across suppliers. Local 3D printing companies often offer the best rates.

^c^ Since some SensorDrop materials must be purchased in packages with more materials than required for a single SensorDrop unit (see minimum order price), the initial material costs for a single SensorDrop unit are $60.77, with costs further reducing to the $25.36 unit cost as more units are developed.*


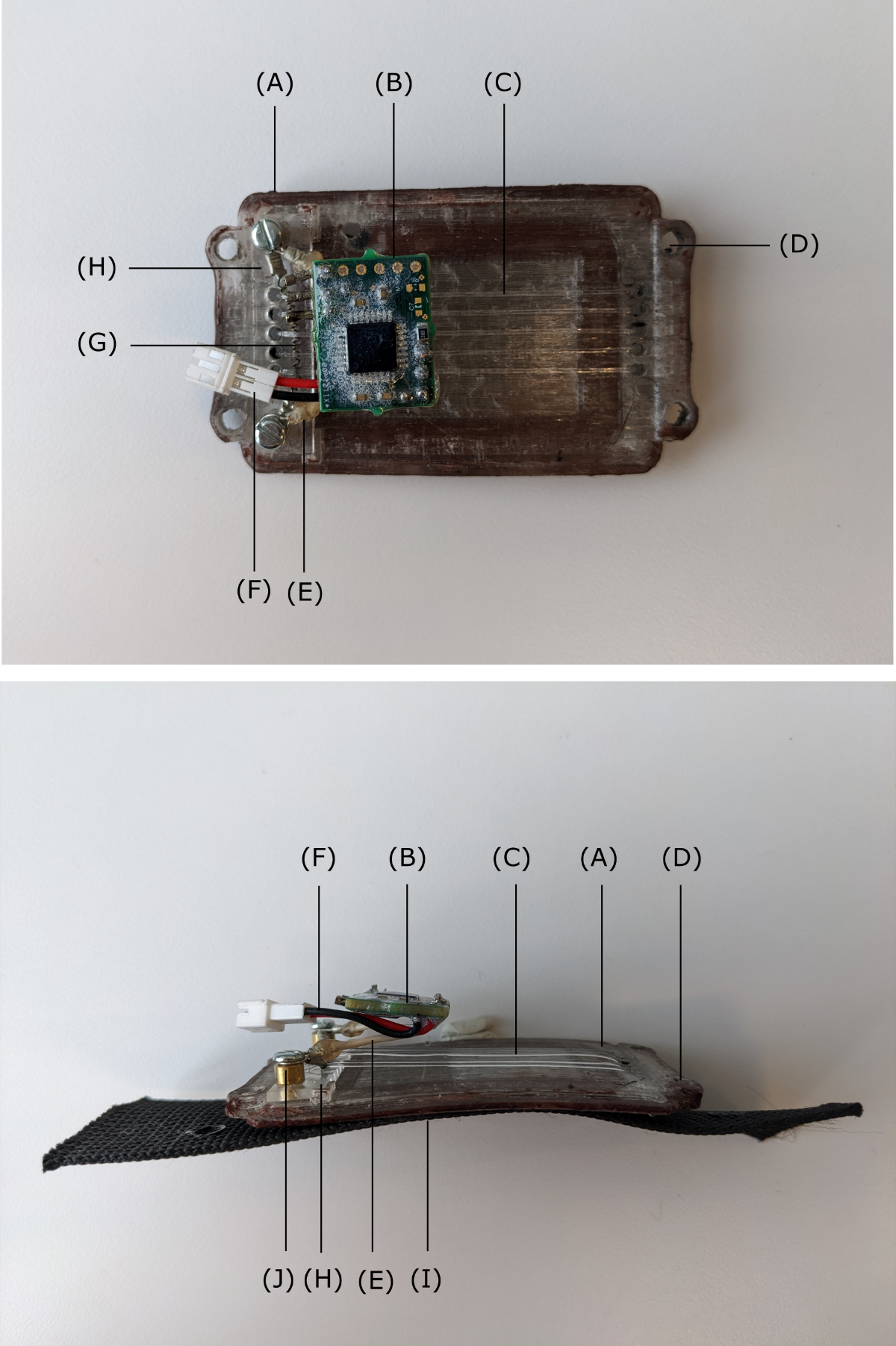


**Figure S1. Detailed summary of key parts of the SensorDrop System**. (A) Drop-off plate. (B) OpenDrop PCB. (C) Nylon line. (D) Holes to screw SensorDrop system onto user-designed housing – one located in each corner. (E) OpenDrop PCB to Nichrome connecting wire. (F) OpenDrop PCB to battery connector. (G) Coiled nichrome element. (H) Acrylic plate to prevent contact between nichrome and the drop-off plate. (I) Nylon webbing. (J) Brass spacers to suspend the nichrome element over the acrylic plate. (K) Hole for VHF transmitter aerial (optional) - can be filled with silicon if no VHF transmitter is used.
