# Supplementary figures and images for: SensorDrop: A system to remotely detach individual sensors from wildlife tracking collars
Source: Ecol Evol. 2023 Jul 4;13(7):e10220. doi: 10.1002/ece3.10220 (PMC10318577; doi:10.1002/ece3.10220)

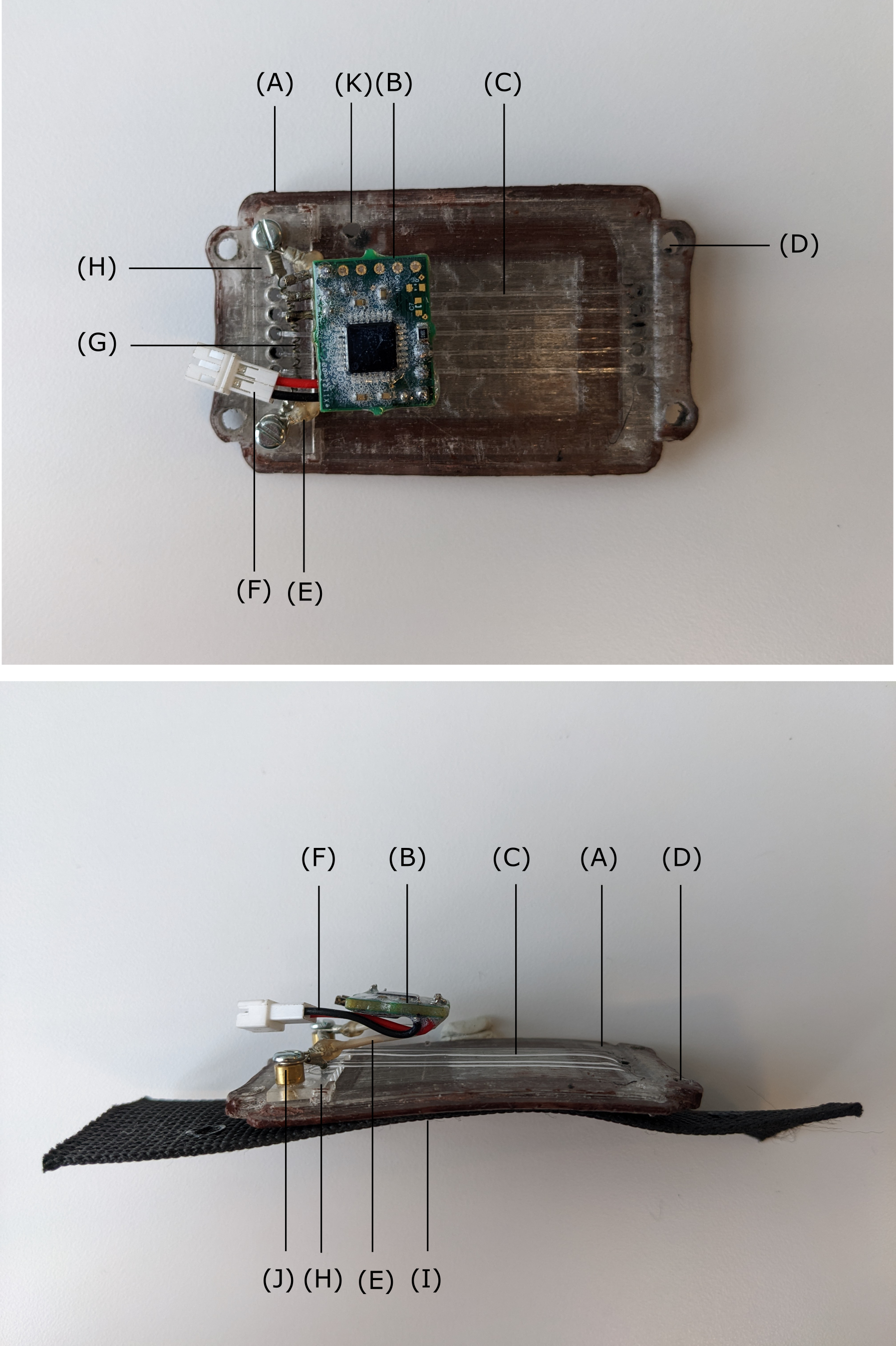

Supplement: Supplementary file 1 — Appendix S1 [file ECE3-13-e10220-s001.zip › Figure S1.png]
